# The Curious Connection Between Insects and Dreams

**DOI:** 10.3390/insects3010001

**Published:** 2011-12-21

**Authors:** Barrett A. Klein

**Affiliations:** Department of Neurobiology and Behavior, Cornell University, Ithaca, NY 14853, USA; E-Mail: barrett@pupating.org

**Keywords:** cultural entomology, ethnoentomology, dreams, dream interpretation, Lewis Carroll, Alice’s Adventures in Wonderland, The Metamorphosis, psychoanalysis, Sigmund Freud, entomophobia

## Abstract

A majority of humans spend their waking hours surrounded by insects, so it should be no surprise that insects also appear in humans’ dreams as we sleep. Dreaming about insects has a peculiar history, marked by our desire to explain a dream’s significance and by the tactic of evoking emotions by injecting insects in dream-related works of art, film, music, and literature. I surveyed a scattered literature for examples of insects in dreams, first from the practices of dream interpretation, psychiatry, and scientific study, then from fictional writings and popular culture, and finally in the etymology of entomology by highlighting insects with dream-inspired Latinate names. A wealth of insects in dreams, as documented clinically and culturally, attests to the perceived relevance of dreams and to the ubiquity of insects in our lives.

## 1. Introduction

Insects are diverse, resourceful, and resilient, serving as symbols of everything from beauty [1] and rebirth [2], to pestilence and evil [3]. Insects pollinate or devour crops [4], contribute to [5,6,7] or wreak havoc on technology [8,9,10], inspire architecture [11,12,13,14] or obliterate it [15,16], and advance human health [4,17,18,19,20] or vector disease [21,22]. Insects inhabit nearly every earthly niche but in the deep marine [23], and can be found among, on, and even inside humans [22,24], and thus it is no surprise that insects have also made their way into our dreams.

Human dreams primarily occur during rapid eye movement sleep (REM), but can also occur during slow wave sleep, typically as non-narrative images [25]. To appreciate the significance of insects’ appearance in dreams, it may be important to understand the functional significance of dreaming. Scientists, by examining the behavior and physiology of dreaming, have attributed various functions to dreaming in humans. David Hartley suggested in the early 19th century that dreams might affect the strength of memories, and recent studies have since linked memory and REM sleep [26]. Crick and Mitchison [27] outlined a scenario of dreams as platforms for selective forgetting, or “reverse learning,” while Revonsuo [28] proposed that dreams simulate threats and allow for us to rehearse threat avoidance. Sigmund Freud claimed that dreams preserve sleep by protecting the sleeper from external stimulation and by allowing us to fulfill our secret wishes harmlessly [29]. Dreams may help to organize our thoughts, solve emotional or intellectual problems, or, acting as a “cinema of the mind,” keep the brain stimulated without having to wake the sleeper [30]. Alternatively, dreams may be meaningless, serving no special function [31]. The search for the function of dreaming and the meaning of dreams occupy traditional biologists, psychologists, anthropologists, as well as philosophers and artists. Dreams featuring insects have been of special interest to medical practitioners, psychotherapists, writers, and others, likely owing to the fact that invertebrates often elicit fear and avoidance responses in humans [32].

Interpreting dreams that include insects is a practice spanning millennia, from the ancient writings of Artemidorus to a spate of freshly posted websites. Writing about insects in dreams is also the stuff of great literature, from Lewis Carroll’s *Alice’s Adventures in Wonderland* to Franz Kafka’s *Die Verwandlung* (*The Metamorphosis*). The association between dreams and insects extends to insect names, some of which stem from dream-related root words. The following, necessarily incomplete survey reports examples of insect dream interpretations, insect dream analysis within psychiatry and science, and of insects in dreams in popular culture, as well as etymological hints of dreams found in insect names.

## 2. Interpretation of Insect Dreams

“The dreams show something further, not suspected or predicted; the bugs have something to teach. They demonstrate the intentions of the natural mind, the undeviating faith of desire, and the urge to survive.”—James Hillman [33].

“Sometimes, it can be difficult to say who is fantasizing more, the dreamer or the dream ‘interpreter’.”—James Horne [30].

Dreams have been analyzed by shamans, soothsayers, and wise men the world over. The list of insects interpreted in dreams is a long one, and the internet is rife with sites claiming to have answers as to the meaning of our insect dreams. With websites like “dreamomania.com [34],” “dreammoods.com [35],” and “myIslamicDream.com [36],” the interpretations are too plentiful to list, but I include examples extracted from these websites in addition to three published books on dreams to illustrate the taxonomic and thematic range of what is available (Table 1).

**Table 1:** Sample of dream interpretations that feature insects, organized by the insects’ common names as they were written in the dream interpretations, what I assume to be their Order and Family affiliations, and the context within which the interpretations were made (e.g., Islamic interpretation of dream in which the dreamer was bitten by a flea). The source for each dream interpretation is indicated by the reference number to the right of each entry.

Insect	ORDER: Family	Context	Interpretation	Ref.
Dragonfly	ODONATA		Change, regeneration, instability, flightiness	[35]
		Eating dragonfly	Consumed by passion even at risk of offending/hurting others’ feelings	[35]
Grasshopper	ORTHOPTERA Acrididae		Freedom, independence, spiritual enlightenment, inability to settle down or commit to decision	[35]
Locust	ORTHOPTERA	Farmers	Devastation of crops	[37]
		All but farmers	Wicked men & women	[37]
		Interp. Gypsies	Extravagance, misfortune, & ephemeral happiness	[38]
			Greed, lack of psychological nourishment, cycles, indecisive	[35]
Cricket	ORTHOPTERA Gryllidae		Introspection	[35]
Katydid	ORTHOPTERA Tettigoniidae		Will miss out on opportunities due to laid back attitude	[35]
Earwig	DERMAPTERA	Interp. Islam	Enemy of the leaders	[36]
Mantis	MANTODEA		Involved in destructive relationship; dreamer behaving deviously	[35]
Termite	ISOPTERA		Attack to your soul or to your being	[35]
Cockroach	BLATTODEA		Uncleanness, longevity, tenacity, renewal; aspect of oneself that needs to be confronted	
Beetle	COLEOPTERA		Destructive influences may be at work; values & beliefs are being compromised	[35]
Dung beetles, cockchafers, firefly immatures	COLEOPTERA Scarabaeidae, Lampyridae	Engage in dirty & ignoble occupations	Success	[37]
		Others	Loss & unemployment	[37]
Scarab	COLEOPTERA Scarabaeidae		Dreamer’s ability to survive, adapt, & change; anxieties about death & aging	[35]
Ladybug	COLEOPTERA Coccinellidae		Beauty & good luck	[35]
Weevil	COLEOPTERA Curculionidae		Losses & deception	[35]
Bed bug	HEMIPTERA Cimicidae		Disgrace	[37]
Louse	PHTHIRAPTERA		Annoyance	[37]
			Frustrations, distress, guilt, feeling unclean	[35]
		Killing lice on dreamer’s body	Release from anxiety & sorrow	[37]
		Cleaning of lice from dreamer’s body	Hope for the release of evils	[37]
		Overabundance on body	Lingering illness	[37]
		Awaken during dream of overabundance on body	Beyond help	[37]
Butterfly	LEPIDOPTERA		Creativity, romance, joy, spirituality, longevity; need to settle down; undergoing transitional phase	
		Interp. Gypsies	Lack of fixed purpose, restlessness, & inconstancy	[38]
Moth	LEPIDOPTERA		Weaknesses, character flaws; unseen irritation may not surface until it is too late	[35]
		Interp. Gypsies	Love affair in which dreamer suffers betrayal	[38]
Caterpillar	LEPIDOPTERA		Have not reached goal	[35]
		Interp. Gypsies	Trouble through secret enemies	[38]
Fly	DIPTERA		Filth, dirtiness, guilt, breakdown of plan, irritating person	[35]
		Interp. Islam	Weak, lowly, & slanderous person	[36]
		Interp. Islam: eating flies, or seeing inside stomach	Earning loathsome money	[36]
		Interp. Islam: inside dreamer’s mouth	Thieves will take refuge or hide in dreamer’s house	[36]
Gnats & dance flies	DIPTERA		Contact with harmful men of evil	[37]
		Innkeepers & wine merchants	Transformation of wine to vinegar	[37]
“Gadfly”	DIPTERA	Interp. Gypsies	Trouble	[38]
Mosquito	DIPTERA Culicidae		Someone has been draining dreamer of energy & resources	[35]
		Interp. Gypsies	Persecution from petty enemies	[38]
		Interp. Islam: enters ear	Blessing, status, authority, or profits	[36]
		Killing mosquitoes	Eventually will overcome obstacles & enjoy happiness & fortune	[35]
Maggot	DIPTERA		Anxieties about death	[35]
Flea	SIPHONAPTERA		Provoked into anger & manipulated to retaliate by someone close	[35]
		Interp. Islam	Allah’s soldiers	[36]
		Bite	Vicious rumors by false friends will slander dreamer	[35]
		Interp. Islam: bite	Earnings	[36]
		Interp. Islam: wounded flea	Weak enemy	[36]
		Interp. Islam: flea’s blood	Receiving money from a lowly person	[36]
Bee	HYMENOPTERA Apidae		Wealth, good luck, harmony, creativity, bliss; hard work will pay off	[35]
		Farmers & beekeepers	Good luck	[37]
		All others	Destroyed by mob or by soldiers	[37]
		Hum	Confusion	[37]
		Sting	Wounds	[37]
		Honey & wax	Sickness	[37]
		Peaceful swarm	Religious vision; holiness of spirits	[39]
Bumble bee	HYMENOPTERA Apidae		Distress & coming problems	[35]
Ant	HYMENOPTERA Formicidae		Diligence, cooperation, increased business, conformity, dissatisfaction with life, restlessness	[35]
		Crawling on dreamer’s body, or sight of winged ants	Death	[37]
		Farmers	Good luck	[38]
		Tradesman	Success	[38]
Wasp	HYMENOPTERA Vespidae		Bodes ill; encounter evil, cruel men	[37]
			Evil, anger, negative feelings	[35]
		Sting	Face envious enemies	[37]
		Interp. Hindus	Unexpected separation	[34]
		Interp. Arabians	Many temptations to resist	[34]
		Interp. Arabians: sting	Grief-filled days	[34]
		Interp. Europeans	Laziness, damage, malice; dangerous attacks on unknown enemies	[34]
		Interp. Europeans: sting	Betrayal by ex-friend (will use knowledge against you)	[34]
		Interp. Europeans: killing wasp	Ability to stand up fearlessly vs. opponents	[34]
Hornet	HYMENOPTERA Vespidae		Trouble & danger ahead	[35]

The earliest known publication on the analysis of dreams is Artemidorus Daldianus’ *Oneirocritica*, a five-volume work with over 3,000 dream accounts written in the 2nd century A.D. Artemidorus viewed insects as symbols of cares and anxieties [37], but wisely cautioned that interpretations of dreams depend on their context, and can vary depending on the dreamer. This context-dependency contributes to a potentially infinite pool of dream interpretations. Take bees for instance; dreaming of bees signifies good luck for farmers and beekeepers, but all others should expect to be destroyed by a mob or by soldiers [37]. Worker ants indicate good luck for farmers, and to the tradesman success [38], but ants crawling on the dreamer’s body portends death [37]. Dreams of locusts signify wicked men and women to all but farmers, who face the devastation of crops [37], but for Gypsies dreams of locusts forecast extravagance, misfortune, and ephemeral happiness [38]. Additionally, Gypsy dream interpretation of a mosquito points to persecution from petty enemies [38], but in Islam when a mosquito enters your ear it denotes blessing, status, authority, or profits [36].

One common contextual category for dream interpretation is the killing of annoying insects. To kill lice on your body signifies a release from anxiety and sorrow, and to clean lice from your body signifies hope for the release of evils [37]. For Europeans, to kill a wasp denotes the dreamer’s ability to stand up fearlessly against opponents [34], and to eat a dragonfly means that you are consumed by passion at the risk of hurting others’ feelings [35]. Interpretations exist for dreams about most of the insect orders, of which the parasitic, biting, and stinging insects are well represented.

## 3. Psychoanalysis and the Scientific Study of Insect Dreams

Insects do not enjoy an entirely positive reputation with humans. In a study of Connecticut residents, the general public viewed most invertebrates “with attitudes of fear, antipathy, and aversion” [32]. “There is a long tradition of hating the bugs,” and in psychoanalysis insects are, for the most part, associated with excrement and anality, plagues, death, evil, negative self-image, *etc*. [33]. James Hillman [33] accounts for humans’ entomophobia in three “fantasies”: (1) due to the tremendous number of insects, dreams of insect invasions can indicate psychotic dissociation, or loss of centralized control, (2) insects in dreams can indicate the psyche’s capacity to conjure up inhuman monstrosities, or (3) insect autonomy threatens us with competition and freedom beyond our control.

A study of children (*n* = 78 four- to six-year-olds) concluded that children’s insect dreams reflect the symbolic feeling of powerlessness attributed to being small [40]. For Martha, the subject of extensive study by several doctors [41], the childhood fear that appeared most frequently in her dreams was that of the cockroach. Garma [42] reasoned that because insects in dreams often represent siblings or a fetus, as Martha matured she experienced guilt-ridden fantasies of performing incestuous acts with her siblings and viewed the cockroach as the symbol of this “dirty” act. Bonime and Bonime [43] proposed that the cockroach could have represented Martha’s sexual ignorance, or even the sperm leading to her mother’s pregnancy. In all analyses, Martha equated the cockroach—that “cold-blooded, unchangeable carrier of disease”—with sex [44]. The object of fear in her dreams had to be faced if the treatment of her phobia was to progress. In order to accomplish this, her therapy demanded the eradication of the cockroach from her dream. After having sprayed the cockroach in her dream, Martha no longer dreamt of cockroaches and subsequently lived with less anxiety.

Another case study was concerned with the dreaming of lice in patients’ hair [45]. Head lice (*Pediculus humanus capitis* de Geer, 1767) are ectoparasites that specialize on human scalp, and louse infestation can have a negative psychological impact on infected hosts [46]. Dreaming of lice infesting one’s head, according to the investigator, reflects the threat to self-representation that arises from treatment. In the eyes of the patients, all of whom were female and had experienced various conflicts, the doctor was infesting their heads with filthy ideas about themselves, which they were forced to acknowledge in the treatment.

Sigmund Freud, in his seminal work *Die Traumdeutung *(*The Interpretation of Dreams*, 1899), recounted a characteristically juicy case study in which insects were featured throughout:

“She recalls that she has two may-beetles in a box which she must set free or else they will suffocate. She opens the box; the beetles are exhausted; one of them flies up out of the opened window, but the other is squashed by the casement as she is closing the window, which someone wants her to do (expression of disgust).”.

[47]

Freud uses this brief dream-thought, dreamt by an elderly lady, to demonstrate condensation—that “The dream is scant, paltry, laconic in comparison to the range and abundance of the dream-thoughts.” The analysis of this dream constitutes pages of anecdotes and interpretations, much of which is peppered with insects. For example, the patient’s daughter had drawn attention to a moth, which fell in her glass of water. Neglecting to rescue the moth, the patient later felt sorry for the drowned insect. Highlighting animal cruelty, Freud included additional anecdotes, such as her daughter’s request for arsenic to produce a butterfly collection, resulting in a lengthy flight of a skewered moth around the room, and chrysalises left to starve. The elderly lady had told her husband “Go hang yourself…” a few hours after reading that a powerful erection sets in when a man is hanged. According to Freud, this shocking utterance disguised her repressed wish for her husband to obtain an erection at all costs. Freud also found it important to note that the patient was aware that the strongest alleged aphrodisiac was cantharides (“Spanish flies”), produced from pulverized beetles [47] belonging to Family Meloidae.

One final example of a case study linked to the clinical analysis of insects in dreams involved the treatment of a forty-year-old engineer by Medard Boss [39]. This three-year study analyzed 823 dreams of the deeply depressed and sexually impotent man. His initial dream images were comprised of only the cold, gray visions of machinery, a dream world void of all living matter. As treatment progressed, the man experienced a “phylogenetic development” of dreams, as was described by Jung C.G. [39]; he first dreamt of a plant, and later of insects, which entered 105 of his dreams. The gray dreams progressively featured reptiles and amphibians, some mammals, and finally humans. Insects were seen to represent one stage in the man’s psychological development and an integral part of his therapy, during which he regained his full potentiality.

The fact that insects are featured in dreams may not necessarily lend special significance to the insect itself. Freud wrote of “displacement [47],” a feature of dreams that can censor something important (e.g., a car wreck) by transferring the central feature of the dream to something unimportant (e.g., an insect on the windshield of the car). If dreams can serve as self-censoring vehicles to suppress important features, then the insect in a dream could merely serve to distract. In psychoanalysis, there is a world of interpretations, and insects have played their share of symbolic roles in dreams. Hillman [33] weaves insects, dreams, religion, and human frailty in his essay “Going Bugs”:

“Our dreams recover what the world forgets. Forgotten pagan polytheism breeds in animal forms. In those animals are the ancient Gods: the Celtic horns and salmon, the Viking bears, the Egyptian pigs and river horses, crocodiles and cats, the Roman wolves and eagles, and Navaho *be’gotcidi.* The old Gods are still there in our dreams—those zoological cathedrals, where there is a mansion for the insects of Beelzebub and Mephistopheles. The animals may go on like Gods, alive and well and unforgotten, in the ikons of our dreams and in the vital obsessions of complexes and symptoms, the little bugs indestructible.”

As with the psychoanalytic studies above, scientific investigations of insects in human dreams often find insects as negative factors in dreams, several examples of which follow.

Dreamlike mentations of insects can occur during sleepwalking or sleep terrors. In one study, Patient 18 saw a crack above her bed in which cockroaches were crawling on dead flesh. Cockroaches fell into her bed and hair, sending her (actually) running from her bed, rubbing her hair [48]. Onset of REM sleep behavior disorder, a disorder in which the dreamer does not face normal muscle paralysis during REM sleep and consequently acts out dreams [49], is coincident with more intense, vivid dreams, such as being chased or attacked by insects [50]. Narcoleptic hallucinations, mostly occurring during sleep onset or offset and suspected to be linked to REM sleep, include reports of “frightening insects” or the “touch” of an insect [51]. Insect-snake phobias were reported more frequently in female students’ dreams than in male students’ dreams in a study conducted across several Canadian universities [52].

## 4. Insects in Dreams in Popular Culture

### 4.1. Art, Film, and Music

**Figure 1:**
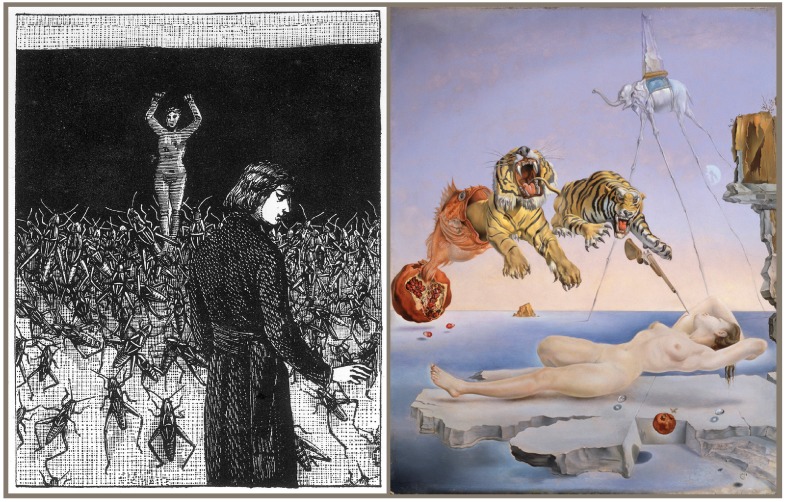
Insects in surrealist dreamscapes. Several dreams depicted in Max Ernst’s book of collages *Rêve d'une petite fille qui voulut entrer au Carmel* (*Dream of a little girl who wanted to join the Carmelite order*, 1930) portray insects, including *… Hoppla! Hoppla! *… (left; reproduced with permission from V.G. Bild-Kunst, Bonn, Germany, 2011); A bee buzzes by a dreamer’s ear in *Dream Caused by the Flight of a Bee Around a Pomegranate a Second Before Awakening* (right; Salvador Dalí, 1944, reproduced with permission from Fundació Gala-Salvador Dalí/V.G. Bild-Kunst, Bonn, Germany, 2011).

Psychoanalysis and dream studies, especially when crawling with insects, are simply too riveting to remain in the office or laboratory. Psychoanalytical studies inspired insect dream paintings by Salvador Dalí and collages by Max Ernst (Figure 1). Filmmakers often conjure up horrific insect dream sequences ripe for psychoanalysis, whether it be of someone turning into a giant cockroach (*Nightmare on Elm Street 4*, 1988), or giving birth to an over-sized maggot (*The Fly*, 1986). In music, insects can also play fearful dream roles (*I Dream of Bees*, by The Bloody Hollies, 2011), but they often appear in more tranquil dream visions (*Dream of the Gryllidae*, album by Yumi Hara and Tony Lowe, 2010; *Dragonfly Dream* by Cylab, 2010; and any number of butterfly or moth dream songs). See Coelho’s writings for a glimpse of how common it is for musicians to dream of insects [53,54].

### 4.2. Literature

Insects also abound in literature, as literal or as metaphorical players [17,55,56]. Insects appear frequently in the works of Shakespeare [57], the poetry of Emily Dickinson [58], and play central roles in Bernard Werber’s *Empire of the Ants*, Daniel Weiss’ *The Roaches Have No King*, Victor Pelevin’s *The Life of Insects*, Aesop’s fable and La Fontaine’s subsequent adaptation of *The Grasshopper and the Ant*, and Kobayashi Issa’s haiku. Insects are critical story components in James Fowler’s *The Collector*, A.S. Byatt’s *Angels & Insects*, Kobo Abe’s *The Woman in the Dunes*, Edgar Allan Poe’s *The Gold-Bug*, and E.O. Wilson’s *Anthill.* And of course there is *Archy*, the cockroach author penned by Don Marquis.

More relevant to this study, insects appear in dreams in literature. Chuangtse (*ca.* 369–286 BCE) famously wrote in *Chuang Tzu* that he dreamt he had turned into a butterfly and could not decide upon waking whether he was a man dreaming of being a butterfly, or a butterfly then dreaming of being a man. Theodore Watts-Dunton, English poet and critic (1832–1914) wrote of hearing a bee and grasshopper in dreams in *The Coming of Love.* Following are a couple of the most celebrated works with insects in dreams in literature.

**Figure 2:**
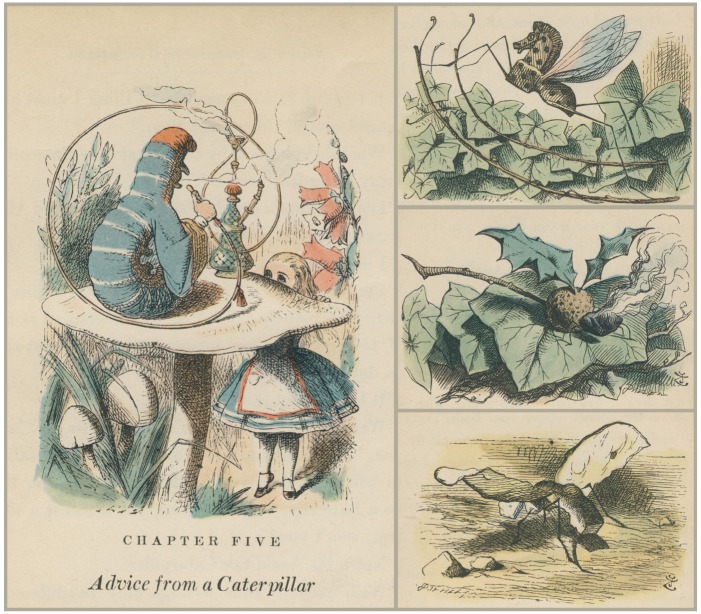
John Tenniel illustrated the Caterpillar (left) for Lewis Carroll’s *Alice’s Adventures in Wonderland*, and the Rocking-horse-fly, the Snap-dragon-fly, and the Bread-and-butter-fly (right) for *Through the Looking-Glass and What Alice Found There*.

**Figure 3:**
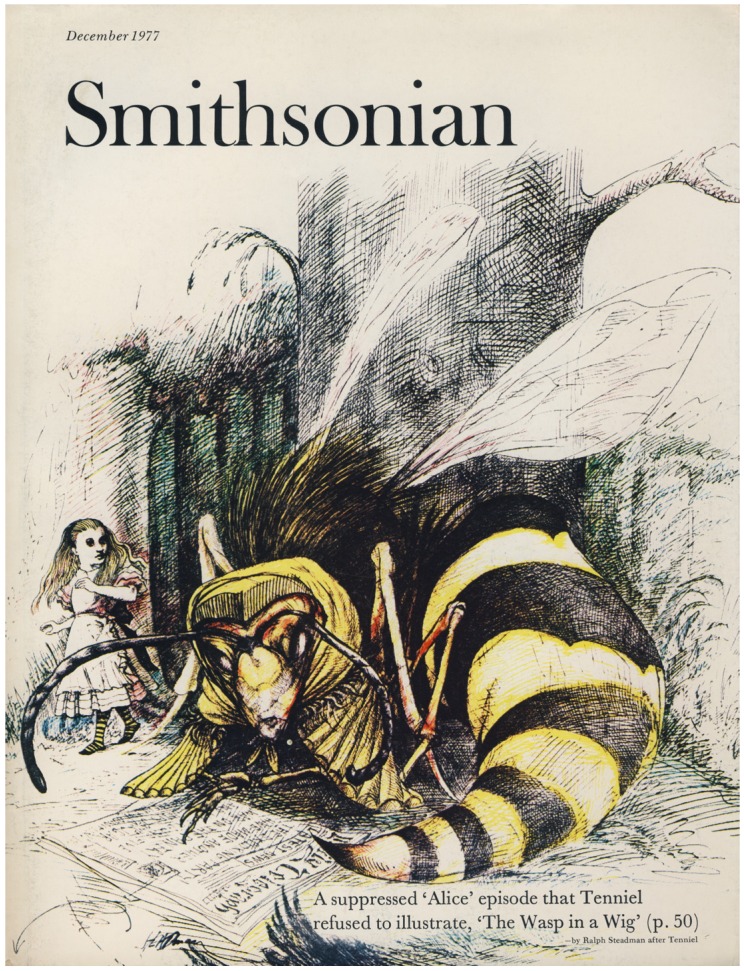
Ralph Steadman produced this cover illustration after John Tenniel, who refused to illustrate the *Wasp in a Wig* episode of *Through the Looking-Glass and What Alice Found There*. Smithsonian magazine printed the unpublished galleys and several attempts to visualize what Tenniel purported was “altogether beyond the appliances of art.” Cover illustration reproduced with permission from Ralph Steadman.

Charles Lutwidge Dodgson, under the pseudonym Lewis Carroll, wrote about the adventures of a girl who dreams of surreal lands with fantastical insects. In *Alice’s Adventures in Wonderland* (1865), a blue, hookah-smoking caterpillar cannot understand why being many different sizes in a day is so confusing to Alice, for the caterpillar is ready to change into a chrysalis and then into a butterfly without a moment’s thought. In *Through the Looking-Glass and What Alice Found There *(1871), Alice travels through a chess game in which she is confronted by a variety of “Looking-Glass insects.” Honey-producing elephants forage like bees, poking their proboscides into cottage-sized flowers. A beetle demands that she travel as luggage during her train ride, and a chicken-sized gnat whispers advice into her ear. The gnat points out the Rocking-horse-fly, which is entirely made of wood, swings from branch to branch, and feeds on sap and sawdust; the Snap-dragon-fly, with body of plum-pudding, head of raisin burning in brandy, and wings of holly-leaves, feeds on frumenty and mince pie and nests in a Christmas box; and finally the Bread-and-butter-fly. “Its wings are thin slices of bread-and-butter, its body is a crust, and its head is a lump of sugar,” and it always dies because it cannot find weak tea with cream, its only source of food [59] (Figure 2). A further episode, *The Wasp in a Wig*, was suppressed by the artist Tenniel because “a wasp in a wig is altogether beyond the appliances of art” [60] (Figure 3). Alice’s adventures have been reinterpreted as films periodically since 1903, including Disney’s 1951 animated feature, as television series, comic books, video games, theme park characters, performances in ballet, opera, and theater, and as a musical album released by Tom Waits.

**Figure 4:**
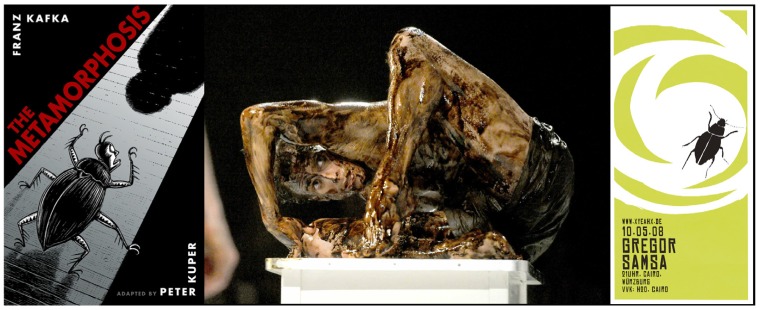
Franz Kafka’s *The Metamorphosis* has crept into many crevices of popular culture. Peter Kuper adapted Kafka’s novella into a graphic novel (left). Edward Watson played Gregor Samsa in Arthur Pita’s stage production in The Linbury Studio, United Kingdom (center). A music band based in Virginia, USA adopted the name Gregor Samsa (right). Cover, photograph, and graphic design reproduced with permission from Peter Kuper [65], Alastair Muir [66], and Oliver Hummel [67].

*Insect Dreams: The Half Life of Gregor Samsa* is Marc Estrin’s 2002 sequel to Franz Kafka’s *Die Verwandlung* (*The Metamorphosis*, 1915), in which the pitiful Gregor Samsa “awoke one morning from uneasy dreams and found himself transformed in his bed into a gigantic insect” [61]. *The Metamorphosis* has found its way into films, cartoons, comic books, stage productions, and references to Gregor Samsa infest popular culture, from the name of music bands to a creature appearing in a video game. Peter Kuper created a graphic novel adaptation in 2003 (Figure 4), and Phillip Glass composed incidental music for theater productions of the story. Samsas Traum (Samsa’s Dream) is a German hard rock band based in Austria, and Gregor Samsa is a band from Virginia with a completely different sound (Figure 4). “*The Metamorphosis* is an allegory which requires interpretation in the same way that a dream requires interpretation if its meaning is to be grasped” [62]. Kafka himself had a great interest in dreams and recorded his own dreams.

Ivan Turgenev, a Russian poet, also recorded his dreams, as in “*The Insect*,” written in the late nineteenth century.

“Suddenly, with a sharp, whirring sound, there flew into the room a big insect, two inches long…. In all of us it excited a sensation of loathing, dread, even terror…. Only one of our party, a pale-faced young man, stared at us all in amazement. He shrugged his shoulders; he smiled, and positively could not conceive what had happened to us, and why we were in such a state of excitement. He himself did not see an insect at all, did not hear the ill-omened whirr of its wings. All at once the insect seemed to stare at him, darted off, and dropping on his head, stung him on the forehead, above the eyes…. The young man feebly groaned and fell dead. The fearful fly flew out at once…. Only then we guessed what it was had visited us.”

[63]

Fyodor Dostoevsky, a contemporary of Turgenev’s, wrote about an insect dream in *The Idiot* (1869):

“I saw the horrible creature, though bitten in two, still wriggling in her mouth and out of its half-crushed body a large quantity of a white fluid, similar to the fluid of a crushed black beetle, was oozing out on to her tongue…. Just then I woke up and the prince came in.”

[64]

### 4.3. Insects as Dream Vectors

Imagined insects enter our dreams, but actual insects can also affect the way we dream, particularly as vectors of diseases that induce fever dreams. Peter Grandbois, reviewer of Rosalind Palermo Stevenson’s *Insect Dreams*, a novella about the life of the 17th century entomologist Maria Sibylla Merian, described Stevenson’s writing like that of a “malarial dream vision.” Alfred Russell Wallace synthesized his inspirational, world-altering work on evolution while suffering from malaria in Indonesia. Do any works of literature, music, and art owe their dreamlike qualities to the fever dreams brought about by a mosquito or by another parasitic insect?

Do arthropods directly affect or can they induce dreaming in sleeping humans? In North America, Chippewa people mimic spider webs when fabricating dream catchers, which are hung from the hoop of a child’s cradle board to capture everything evil [68], while the Blackfeet speak of butterflies as bringing dreams to sleeping humans:

“You know that it is the butterfly who brings us our dreams—who brings the news to us when we are asleep. Have you never heard a man say, when he sees a butterfly fluttering over the prairie, ‘There is a little fellow flying about that is going to bring news to some one tonight’? Or have you not heard a person say after night, as the fire burns low and the people begin to make up their beds about the lodge, ‘Well, let us go to bed and see what news the butterfly will bring’ [69] ?”

Apart from bringing dreams, butterflies are also said to facilitate sleep onset. Blackfeet women tie a cross symbolizing a butterfly in a baby’s hair and sing a lullaby asking the butterfly to fly about in the hope it will induce sleep. Why or how would a butterfly bring sleep? Grinnell [69] reported hearing statements that the butterfly is “soft and pretty and moves gently and that if you look at it for a long time you will go to sleep.”

## 5. Insects Named after Sleep and Dreaming

Occasionally, the very nomenclature ascribed to insects connotes dreaming. *Enypnium quadripunctatum* Kertesz, 1914 is a fly named with the Greek root enypnion, or dream. *Oniromyia pachycerata* Bigot, 1892 is another species of fly, using the Greek root oneiros, or dream vision. Beetles following such a dream-inspired nomenclature include *Ellopia somniaria* Hulst, 1886, with the Latin word for dream (somnium), and *Comatopselaphus parcepunctatus* Raffray, 1904 with the Greek root for putting to sleep (koimistikos). Koimistikos was also used in naming the grasshopper *Cephalocoema lineata* Brunner von Wattenwyl, 1890 (*Carphoproscopialancea* Burmeister, 1880). Finally, the Greek root geleches, or sleeping on the ground, was incorporated into the genus name of the butterfly *Gelechia notatella* Hübner, 1813 [70,71]. Scientific names often reflect either the morphology or behavior of the named subject, and for an entomologist to incorporate sleep- and dream-related root words into their subjects’ names may reflect the insects’ dreamlike beauty or their somnolent behavior. There is ample evidence to suggest that insects sleep [72,73,74,75,76,77,78,79,80,81,82,83,84,85,86,87,88,89,90], but the study of insect dreaming is the topic of future study.

## 6. Conclusions

Insects that are so numerous and successful in our awake state also appear to prosper in our dreams. Insects in dreams are frequently objects of fear and dread, sometimes prompting psychoanalysis and medical investigation. The connection between insects and dreams is not limited to psychoses and nightmares, however. The connection survives in cultural and historical flourishes of dream interpretation, art, music, film, and literary dream sequences, and creative species descriptions. Insects and dreams each affect our daily lives and together have the capacity to inspire us in the most curious of ways.
